# Supplementary material for: The interactome of intact mitochondria by cross-linking mass spectrometry provides evidence for coexisting respiratory supercomplexes
Source: Mol Cell Proteomics. 2017 Dec 8;17(2):216–32. doi: 10.1074/mcp.RA117.000470 (PMC5795388; doi:10.1074/mcp.RA117.000470)
Supplement: Supplemental Data [file supp_RA117.000470_133922_0_supp_23978_fzffwf.zip › spectra_annotation/mito_spectra_annotation/309-1-4-1-1-1.pdf]

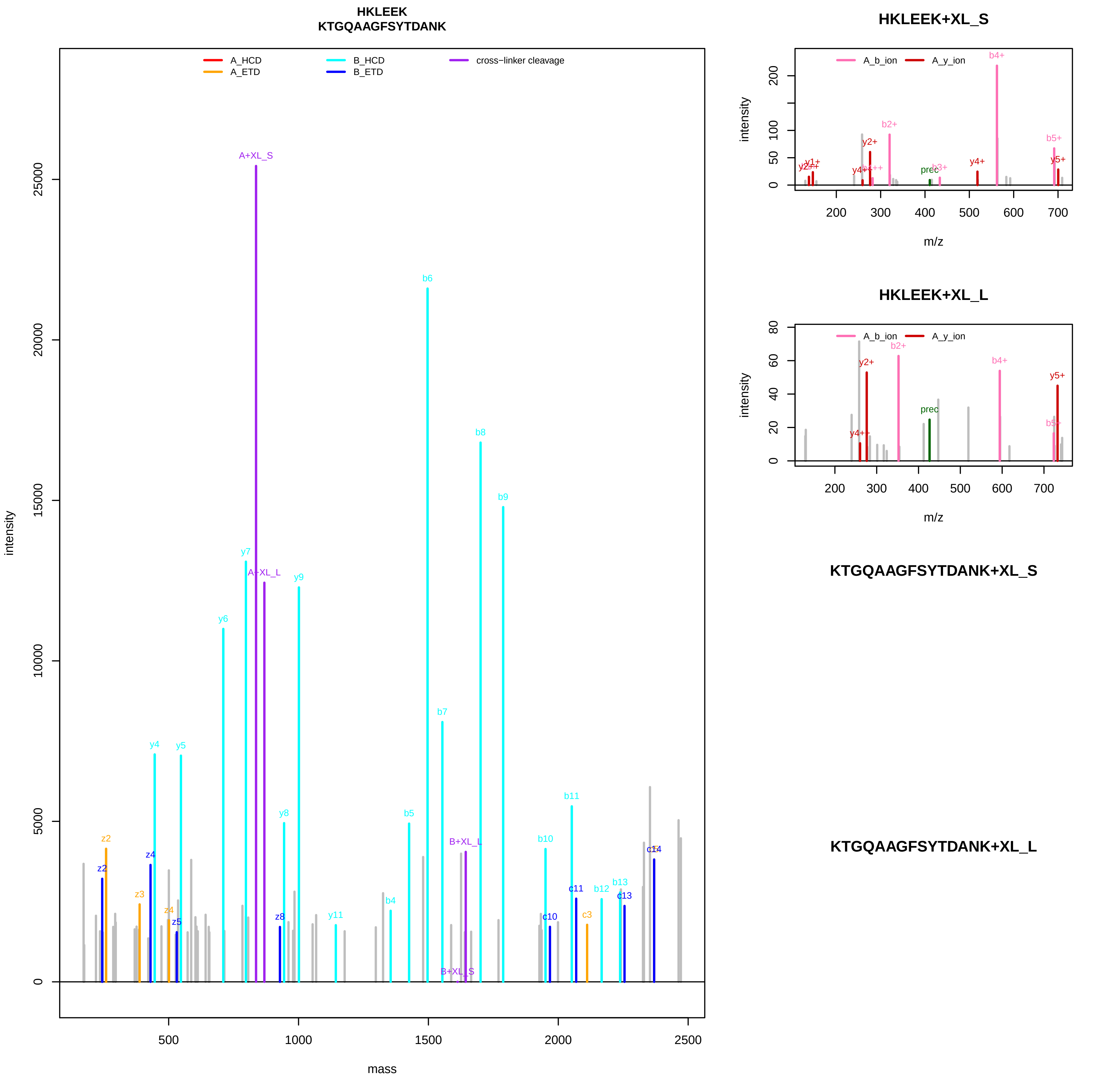
HKLEEK
KTGQAAGFSYTDANK
A_HCD
A_ETD
B_HCD
B_ETD
cross−linker cleavage
intensity
mass
A+XL_S
A+XL_L
B+XL_L
B+XL_S
b4
b5
b6
b7
b8
b9
b10
b11
b12
b13
y4
y5
y6
y7
y8
y9
y11
z2
z3
z4
z5
z8
c3
c10
c11
c13
c14
HKLEEK+XL_S
A_b_ion
A_y_ion
m/z
b2+
b3+
b4+
b5+
y1+
y2+
y4+
y5+
prec
HKLEEK+XL_L
KTGQAAGFSYTDANK+XL_S
KTGQAAGFSYTDANK+XL_L
